# 2.5D and 3D segmentation of brain metastases with deep learning on multinational MRI data

**DOI:** 10.3389/fninf.2022.1056068

**Published:** 2023-01-18

**Authors:** Jon André Ottesen, Darvin Yi, Elizabeth Tong, Michael Iv, Anna Latysheva, Cathrine Saxhaug, Kari Dolven Jacobsen, Åslaug Helland, Kyrre Eeg Emblem, Daniel L. Rubin, Atle Bjørnerud, Greg Zaharchuk, Endre Grøvik

**Affiliations:** ^1^CRAI, Division of Radiology and Nuclear Medicine, Department of Physics and Computational Radiology, Oslo University Hospital, Oslo, Norway; ^2^Department of Physics, Faculty of Mathematics and Natural Sciences, University of Oslo, Oslo, Norway; ^3^Department of Ophthalmology, University of Illinois, Chicago, IL, United States; ^4^Department of Radiology, Stanford University, Stanford, CA, United States; ^5^Division of Radiology and Nuclear Medicine, Oslo University Hospital, Oslo, Norway; ^6^Department of Oncology, Oslo University Hospital, Oslo, Norway; ^7^Division of Radiology and Nuclear Medicine, Department of Physics and Computational Radiology, Oslo University Hospital, Oslo, Norway; ^8^Department of Biomedical Data Science, Stanford University, Stanford, CA, United States; ^9^Department of Radiology, Ålesund Hospital, Møre og Romsdal Hospital Trust, Ålesund, Norway; ^10^Department of Physics, Norwegian University of Science and Technology, Trondheim, Norway

**Keywords:** segmentation, brain metastases, deep learning, MRI, 2.5D, 3D

## Abstract

**Introduction:**

Management of patients with brain metastases is often based on manual lesion detection and segmentation by an expert reader. This is a time- and labor-intensive process, and to that end, this work proposes an end-to-end deep learning segmentation network for a varying number of available MRI available sequences.

**Methods:**

We adapt and evaluate a 2.5D and a 3D convolution neural network trained and tested on a retrospective multinational study from two independent centers, in addition, nnU-Net was adapted as a comparative benchmark. Segmentation and detection performance was evaluated by: (1) the dice similarity coefficient, (2) a per-metastases and the average detection sensitivity, and (3) the number of false positives.

**Results:**

The 2.5D and 3D models achieved similar results, albeit the 2.5D model had better detection rate, whereas the 3D model had fewer false positive predictions, and nnU-Net had fewest false positives, but with the lowest detection rate. On MRI data from center 1, the 2.5D, 3D, and nnU-Net detected 79%, 71%, and 65% of all metastases; had an average per patient sensitivity of 0.88, 0.84, and 0.76; and had on average 6.2, 3.2, and 1.7 false positive predictions per patient, respectively. For center 2, the 2.5D, 3D, and nnU-Net detected 88%, 86%, and 78% of all metastases; had an average per patient sensitivity of 0.92, 0.91, and 0.85; and had on average 1.0, 0.4, and 0.1 false positive predictions per patient, respectively.

**Discussion/Conclusion:**

Our results show that deep learning can yield highly accurate segmentations of brain metastases with few false positives in multinational data, but the accuracy degrades for metastases with an area smaller than 0.4 cm^2^.

## 1. Introduction

Brain metastases are the most common intracranial tumors among both primary and secondary tumors (Johnson and Young, 1996). Contrast enhanced magnetic resonance imaging (MRI) is routinely used for diagnosis and assessment of treatment response, as well as determining the lesion size and multiplicity (Takei et al., 2016). Manual detection and delineation of brain tumors on high resolution multisequence 3D MR image-series for clinical assessment such as radiation therapy is time- and labor-intensive. To this end, automated delineation and detection of brain tumors have been an active avenue of research to ease the burden on radiologists and improve treatment planning (Bauer et al., 2013).

Current regimen for brain metastases treatment planning includes stereotactic radiotherapy, whole brain radiotherapy, surgical excursion and chemotherapy where the number of metastases, location and unidimensional measurements are used for treatment planning (Lin and DeAngelis, 2015). However, studies have shown that volumetric assessment show less intra- and interobserver variability compared to unidimensional measurements (Bauknecht et al., 2010). Nonetheless, given that volumetric analysis of brain metastases would add complexity, costs and workload, this approach is not universally endorsed by expert groups (Lin et al., 2015). To this end, robust automated delineation of brain metastases that can generalize to different clinical protocols and centers is imperative to facilitate volumetric analysis of brain metastases while avoiding observer variability.

Recent advances in deep learning have ushered a new gold-standard in computer-based learning. Traditional deep learning methods include image classification (Russakovsky et al., 2014; He et al., 2016; Krizhevsky et al., 2017; Tan and Le, 2019; Dosovitskiy et al., 2020), segmentation (Ronneberger et al., 2015) and object detection (Girshick et al., 2013; Redmon et al., 2016). In medical imaging, several deep learning methods have been developed and used to automate tedious and time-consuming tasks, perhaps most clearly exemplified in detection and segmentation of pathology (Ronneberger et al., 2015; Milletari et al., 2016; Kamnitsas et al., 2017; Isensee et al., 2020a).

Specifically, deep learning methods have been successfully developed and tested for primary brain tumors, thanks in part to the publicly available BraTS dataset (Menze et al., 2015). Recent studies on in-house data have also shown great promise in using deep learning for detection and segmentation of brain metastases (Charron et al., 2018; Bousabarah et al., 2020; Grøvik et al., 2020; Xue et al., 2020; Zhang et al., 2020; Jünger et al., 2021), with DeepMedic (Kamnitsas et al., 2017) and U-Net (Ronneberger et al., 2015) like architectures commonly used as the deep learning method. However, common challenges raised are high rates of false positive and inaccurate segmentation of smaller lesions (Charron et al., 2018; Bousabarah et al., 2020; Dikici et al., 2020; Grøvik et al., 2020, 2021; Zhang et al., 2020; Zhou et al., 2020a). In addition, multiple studies show a high degree of dataset homogeneity due to the exclusion of patients not receiving stereotactic radiosurgery or single center studies (Cao et al., 2021; Hsu et al., 2021; Jünger et al., 2021; Rudie et al., 2021).

In this study, we implemented and evaluated 2.5D and 3D models for brain metastases segmentation that were tested on multinational data with different clinical protocols and a varying number of input MRI sequences. The high-resolution network for 2.5D and 3D segmentation (Wang et al., 2019) was adopted in combination with mixup augmentation (Zhang et al., 2017), and deep supervision (Wang et al., 2015). We demonstrate that the proposed 2.5D and 3D deep learning-based segmentation models can successfully be used for segmentation on two separate clinical protocols, whilst reducing the number of false positives previously reported for both cohorts without reducing the number of successfully detected metastases (Grøvik et al., 2020, 2021; Yi et al., 2021). Method performance was evaluated by adopting the nnU-Net (Isensee et al., 2020a) framework as a comparative benchmark. The model weights for the 2.5D and 3D networks have been made publicly available.^1^

## 2. Materials and methods

### 2.1. Multinational dataset information

This retrospective multinational study was approved by the Regional Medical Ethics Committee for Oslo University Hospital (OUH) and the Institutional Review Board at Stanford University. The OUH dataset (TREATMENT; clinicaltrials.gov identifier: NCT03458455) consisted of 65 patients eligible for stereotactic radiotherapy with pre- and post-contrast T1-weighted fast spin echo (SPACE) and a 3D fluid-attenuated inversion recovery (FLAIR) image-series. The Stanford dataset consisted of 156 patients that underwent imaging with a 3D inversion recovery fast spoiled gradient echo (BRAVO), pre- and post-contrast T1-weighted fast spin echo (CUBE), and a 3D FLAIR. Additional scan parameters and patient demographics are given in Tables 1, 2, respectively.

**Table 1:** An overview of the MRI sequence related parameters.

	**3D BRAVO**	**3D T1 CUBE/SPACE**	**3D FLAIR**
**Stanford cohort**			
TR^*^ (ms)	12.02/8.24	550/602	6,000
TE^*^ (ms)	5.05/3.24	9.54/12.72	119/136
Flip angle^*^ (deg)	20/13	90	90
FOV (mm^2^)	240 × 240	250 × 250	250 × 250
Inversion time^*^ (ms)	300/400	-	1,880/1,700
Acquisition matrix	256 × 256	256 × 256	256 × 256
Slice thickness (mm)	1	1	1–1.6
# of slices	160	270–320	270–320
Acquisition plane	Axial	Sagittal	Sagittal
**OUH Cohort**			
TR (ms)		700	5,000
TE (ms)		12	387
Flip angle		120	120
FOV (mm^2^)		230 × 230	230 × 230
Inversion time (ms)		-	1,800
Acquisition matrix		256 × 256	256 × 256
Slice thickness (mm)		0.9	0.9
# of slices		192	208
Acquisition plane		Sagittal	Sagittal

TR, repetition time; TE, echo time; FOV, field-of-view; BRAVO, T1-weighted inversion recovery prepped fast spoiled gradient-echo; CUBE, T1-weighted fast spin-echo; FLAIR, fluid attenuated inversion recovery. Varying parametric values are denoted by asterisk (^*^) notation and ‘/'.

**Table 2:** Patient demographics for both hospital cohorts and the number of patients with a given number of metastases.

**Demographics**	**OUH cohort**	**Stanford cohort**
Gender	35 F/30 M	105 F/51 M
Age range	32–86	32–92
**Primary cancer**		
Lung	45	99
Breast	20	33
Skin/melanoma	–	7
Genitourinary	–	7
Gastrointestinal	–	5
Miscellaneous	–	5
# of metastases	151	860
≤ 3	54	58
4–10	10	46
≥10	1	52

The OUH ground truth annotations were established by two working radiologists with 5 and 14 years of experience. For the Stanford dataset, ground truth annotations were established by two neuroradiologists with 8 and 2 years of experience. Twenty-six of the original ground truth annotations were later revised and edited by the working neuroradiologist with now 5 years of experience.

**Figure 1:**
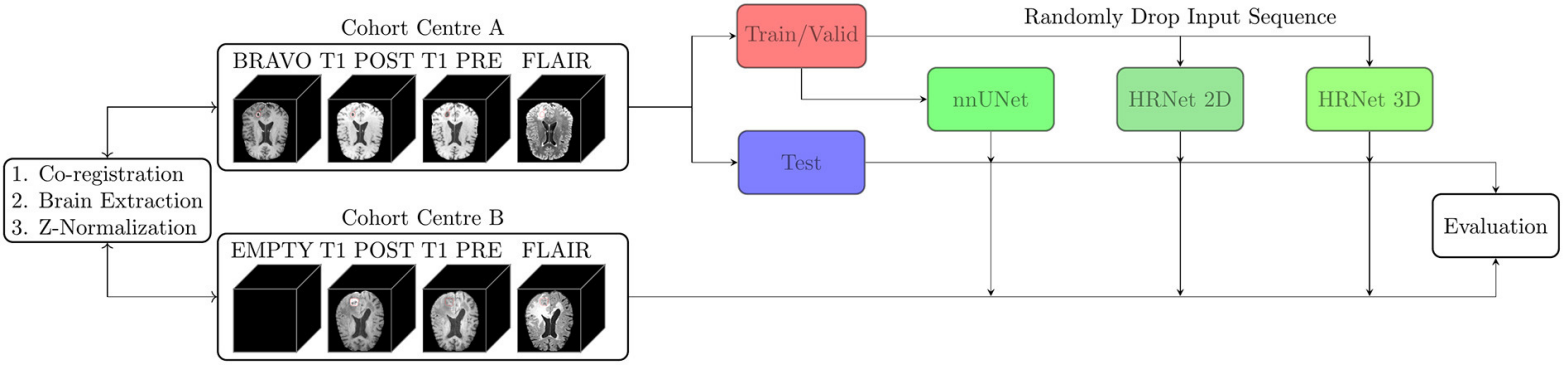
Two patient cohorts from different institutions (A and B) were annotated by working radiologists, coregistered, and brain extracted. Cohort A was divided into a training/validation set and a test set, whilst cohort B was kept as an independent test set. Three models were trained on the training set from cohort A, and evaluated on the test set from cohort A and cohort B. Two of the models were trained with input-level dropout to allow for a variable number of input MR sequences.

To test model generalizability and robustness, model training was performed by only including data from the Stanford cohort. Hundred patients were randomly selected for the training dataset, 10 patients were randomly selected for model validation, and the remaining 51 patients were used for model testing. Moreover, all 65 patients from the OUH were used for model evaluation. In total, 860 and 151 metastases from the Stanford and OUH cohorts were used for model evaluation, respectively. A flowchart of the study design is illustrated in Figure 1, where cohort A and B represent the Stanford and OUH cohort, respectively.

### 2.2. Model architecture

**Figure 2:**
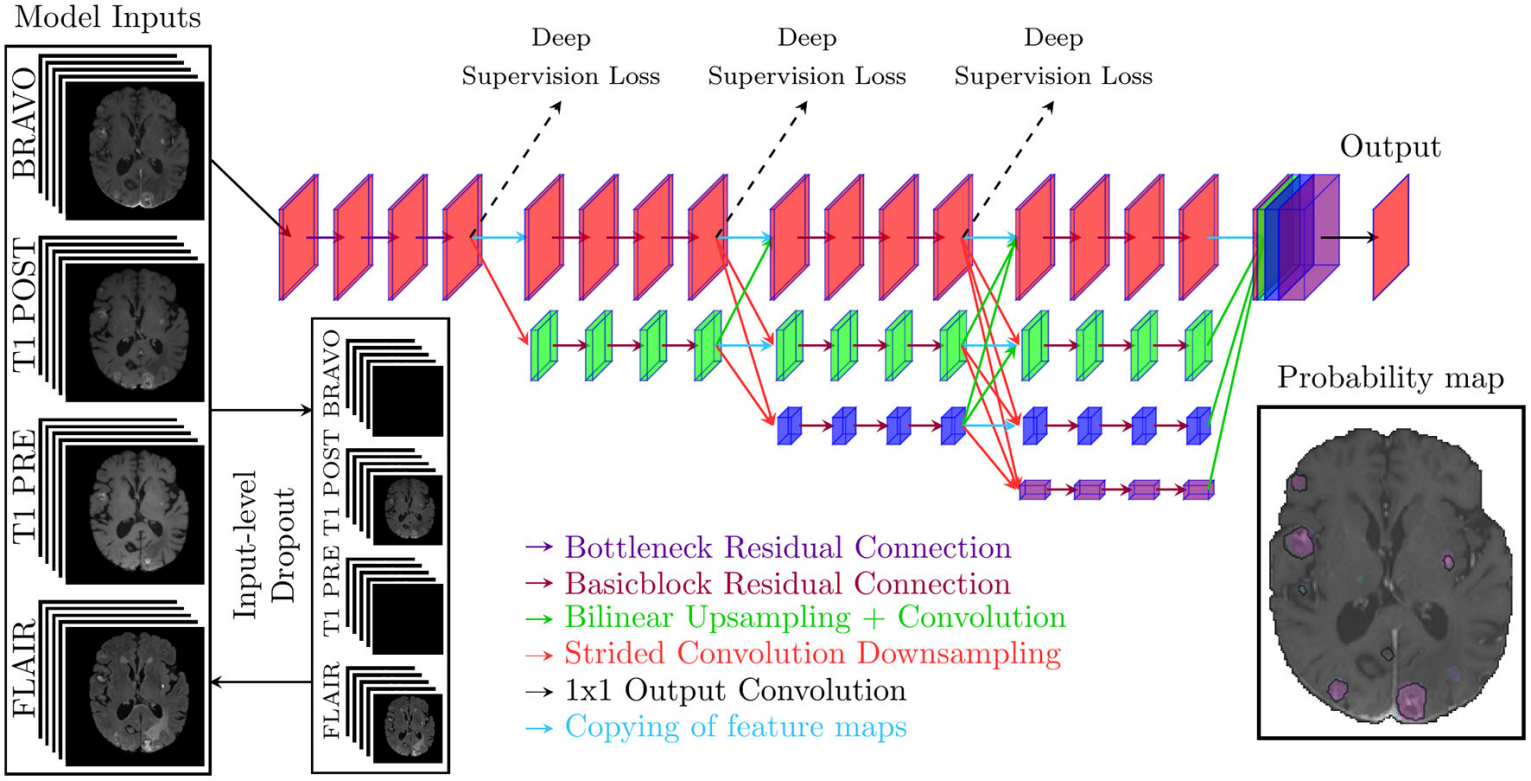
The general model architecture that takes either five slices from one to four sequences as inputs for the 2.5D model or a volume from one to four sequences for the 3D model. For the 2.5D model, the inputs are upscaled with bilinear upscaling before downsampling through a convolutional operation. The model prediction is a probability map between 0 and 1.

In this study, two deep learning models were implemented and tested: one 2.5D architecture for slice-wise segmentation and a 3D architecture for volume-wise segmentation. Both the 2.5D and 3D networks are based on the high-resolution net V2 (HRNetV2) (Wang et al., 2019). The 2.5D and 3D variants were adopted to evaluate whether a 2D or 3D segmentation approach is best suited for brain metastases segmentation. The general model architecture is illustrated in Figure 2. Because previous studies have raised the issue of reduced performance for small metastases, all model inputs for the 2.5D model were upscaled by two-fold bilinear upscaling followed by a convolutional operation for resolution reduction. This additional upscaling operation showed improved performance during an initial testing phase but was not performed on the 3D model variant due to memory constraints. In addition to the two architectures above, the self-configurable nnU-Net was adopted as a comparative benchmark since the nnU-Net pipeline has previously shown state-of-the-art performance in medical image segmentation tasks (Isensee et al., 2020b).

HRNetV2 was chosen as the reference model because the architecture combined with object contextual representation (Yuan et al., 2019) has previously archived state-of-the-art performance on the cityscape dataset (Cordts et al., 2016). In addition, H2NF-Net—a HRNetV2 like model achieved second place in the BraTS challenge 2020 (Jia et al., 2020). Unlike H2NF-Net, we opted to use a U-Net like decoder for the 3D network to decrease memory constraints during training.

### 2.3. Preprocessing

Every MRI sequence was coregistered to either the BRAVO sequence from the Stanford patient-cohort or the T1-weighted post-contrast image-series from the OUH patient-cohort. Coregistration was performed using the nordicICE software package (NordicImagingLab, Bergen, Norway) by maximizing normalized mutual information. Brain extraction was performed using the deep learning method HD-BET (Isensee et al., 2019) on the T1-weighted pre-contrast image-series and the resulting brain masks were propagated to all other image-series. Every MRI sequence was oriented in the left posterior superior direction after coregistration.

The brain extracted image-series were (if necessary) rescaled to a voxel size of 0.9375 × 0.9375 × 1 mm by trilinear interpolation, and the corresponding ground truth annotations were interpolated by nearest interpolation. Minimal artifacts were encountered in the interpolation process of the brain extracted image-series.

All image-series were standardized with a mean of zero and standard deviation of one. The standardization processes excluded all non-brain extracted voxels, i.e., all excluded voxels were zero-valued.

### 2.4. Training

The 2.5D model was trained for 150 epochs, and a single epoch contained ~12,000 randomly selected brain slices; by extention, each epoch contained ~12,000 training examples. In the selection process, image slices with metastases were weighted ten-fold compared to non-metastases slices (Grøvik et al., 2020). AdamW (Loshchilov and Hutter, 2017) was used as the optimizer with an initial learning rate of 5 × 10^−4^, with an initial warm up period of 10 epochs followed by the cosine annealing learning rate scheduler (Loshchilov and Hutter, 2016), weight decay of 0.01, Amsgrad enabled (Reddi et al., 2018), and a batch size of 16. After every epoch, the model was evaluated on all non-zero and non-augmented slices from the 10 validation patients.

The 3D model was trained for 1000 epochs, and a single epoch contained 95 volumes; by extension, each epoch contained 95 training examples. AdamW (Loshchilov and Hutter, 2017) was used with a learning rate of 5·10ˆ(−3) with an initial linear warm up period for the first 50 epochs, afterwards cosine annealing was used as the learning rate scheduler (Loshchilov and Hutter, 2016), and a batch size of 2. After every epoch, the model was evaluated on non-augmented patches from the 10 validation patients.

All data augmentation performed is detailed in Table 3 with the corresponding probability for said augmentation. To handle any missing input MRI image-series and enforce robust models that generalize to multiple clinical protocols, input-level dropout (Grøvik et al., 2021) was used during training. All image-series had a 25% probability of being omitted and if all image-series were omitted, one sequence was randomly selected to be included. Data augmentation was performed through the Monai framework (Consortium, 2022) except mixup (Zhang et al., 2017) and input-level dropout (Grøvik et al., 2021). Note that mixup was not performed for the 3D model as initial testing showed a decrease in performance.

**Table 3:** An overview of the augmentation methods used, the network that used said augmentation method and the corresponding probability for their use.

**Method**	**Probability**	**2.5D network**	**3D network**
Mixup	100%	Yes	No
InputLevelDropout	25% pr sequence	Yes	Yes
Vertical flipping	25%	Yes	Yes
Horizontal flipping	25%	Yes	Yes
Random head rotation	25%	Yes	Yes
Mean intensity shift	10%	Yes	Yes
Std intensity shift	10%	Yes	Yes
Random contrast change	10%	Yes	Yes
Random histogram intensity shift	10%	Yes	Yes
Random head alignment	10%	No	Yes

All augmentation was performed courtesy of the Monai (Consortium, 2022) framework except Input-level dropout (Grøvik et al., 2021) and mixup (Zhang et al., 2017).

Network optimization was performed by minimization of a compound loss of equal weighting between the Focal Tversky loss (Salehi et al., 2017) and a weighted binary cross entropy (BCE) loss function. Compound loss was chosen since it has been shown to improve the robustness of the segmentation (Ma et al., 2021), and compound loss is used by the state-of-the-art nnU-Net (Isensee et al., 2020a). Unlike nnU-Net, the focal Tversky loss function was used instead of the dice loss to emphasize hard examples and handle class imbalance (Abraham and Khan, 2018). In weighted BCE, every segmented ground truth voxel was weighed ten-fold compared to non-segmented voxels. The loss function used is given by


(1) $$\begin{array}{lll} Loss(y,y\;ˆ\;) & = & FocalTversky(y,y\;ˆ\;)+BCE(y,y\;ˆ\;)\\ & + & \beta \cdot y\cdot BCE(y,y\;ˆ\;), \end{array}$$

where β = 10 is the ten-fold weighted segmented voxels. Focal Tversky loss was used to emphasize the detection of true positives, i.e., metastases. Note, batchwise focal Tversky was used for 2.5D segmentation, whilst imagewise focal Tversky was used for 3D segmentation.

Memory consumption was reduced through mixed precision training, and all slices were randomly cropped once per input to a patch size of 176 × 176 or 128 × 128 × 128 while maximizing the inclusion of brain tissue by centering the cropping around the central region of the brain. The total training time was approximately 20 and 75 hours for the 2.5D and 3D networks, respectively. Both models were trained on a Nvidia A100 with 40GB of memory, whereas the nnU-Net was trained on a RTX 3090 with default settings and a 5-fold cross validation training scheme. Note, input-level dropout was not implemented into the nnU-Net pipeline, for that reason, two versions of nn-UNet were trained: one version trained with the BRAVO sequence and one version trained without the BRAVO sequence.

### 2.5. Evaluation

Segmentation performance was evaluated using a slice-wise dice similarity coefficient given by


(2) $$\begin{array}{l} Dice=\left(2\cdot TP\right)/\left(2\cdot TP+FP+FN\right), \end{array}$$

where TP is the number of correctly predicted metastases voxels, FP is the number of missed metastases voxels and FN is the number of erroneously predicted non-metastases voxels. All correctly predicted zero-slices were given a perfect dice score of 1.

Detection performance was evaluated by the rate of metastases detection (sensitivity), the mean per patient sensitivity and the total number of false positive metastases. The metastases sensitivity is given by


(3) $$\begin{array}{l} \text{“}Sensitivity\text{”}=\text{“}Detected\;Lesions\text{”}\;/\text{“}Total\;Lesions\text{”}, \end{array}$$

where a lesion is defined as the fully connected 3D region of voxels. A metastasis was defined as detected if the prediction had a 10% or larger overlap with a 3D fully connected region in the ground truth annotations. Only connected voxel regions in the ground truth larger than a single voxel were considered as a metastasis when evaluating the detection sensitivity. A prediction was labeled as a false positive if a 3D fully connected prediction had less than 10% overlap with the ground truth annotations. All non-connected single voxel predicted regions were omitted when the model sensitivity, per patient sensitivity, and false positives were estimated. Note that non-connected single voxel predicted regions were not omitted when calculating the dice similarity coefficient.

Input-level dropout was not performed during inference. However, since the OUH cohort lacked the BRAVO sequence, only the T1-weighted pre/post-contrast and FLAIR were used during inference. The probability threshold was chosen to maximize the dice similarity coefficient on the validation dataset for the respective models.

## 3. Results

The dice similarity coefficient, sensitivity, the average sensitivity per patient, and the mean number of false positive predictions per patient for the Stanford and OUH cohorts are given in Table 4. A threshold of 0.98 and 0.99 was used for the predictions from the 2.5D and 3D models, respectively. The dice coefficient and sensitivity were higher for the OUH cohort compared to the Stanford cohort, while achieving a reduced rate of false positives for all three models.

**Table 4:** The dice similarity coefficient, metastases detection sensitivity, the average metastases detection sensitivity per patient, and the average number of false positive metastases per patient with the corresponding standard deviation for the Stanford and OUH cohorts.

**Cohort**	**Model**	**Dice**	**Sensitivity**	**Sensitivity per patient**	**False positives per patient**
Stanford	2.5D	0.84 ± 0.13	0.79	0.88 ± 0.19	6.2 ± 11.4
Stanford	3D	0.84 ± 0.13	0.71	0.84 ± 0.18	3.2 ± 6.5
Stanford	nnU-Net	0.85 ± 0.13	0.65	0.76 ± 0.26	1.7 ± 3.5
OUH	2.5D	0.93 ± 0.04	0.88	0.92 ± 0.15	1.0 ± 1.1
OUH	3D	0.93 ± 0.04	0.86	0.91 ± 0.17	0.4 ± 0.7
OUH	nnU-Net	0.94 ± 0.05	0.78	0.85 ± 0.23	0.1 ± 0.4

Only non-single voxel metastases were counted as metastases for the sensitivity, in contrast, all voxels were considered when evaluating the dice similarity coefficient and the number of false positives.

The fraction of the total number of metastases detected, i.e., sensitivity was 0.79, 0.71, and 0.65 for the 2.5D, 3D, and nnU-Net, respectively. In contrast, the per patient sensitivity was 0.88, 0.84, and 0.76; this variation is mainly caused by a single patient outlier with 153 metastases where only 56, 46 and 41 metastases were successfully detected. In total: 676, 607, and 556 metastases out of 860 metastases were successfully detected by the 2.5D, 3D, and nnU-Net, respectively.

For the independent cohort (OUH cohort), the sensitivity was 0.88, 0.86, and 0.78 with a corresponding sensitivity per patient of 0.92, 0.91 and 0.85 for the 2.5D, 3D, and nnU-Net, respectively. Unlike the Stanford cohort, there wasn't any notable outlier due to fewer metastases per patient. In total: 133, 130, and 118 metastases out of 151 metastases were successfully detected by the 2.5D, 3D, and nnU-Net, respectively.

**Figure 3:**
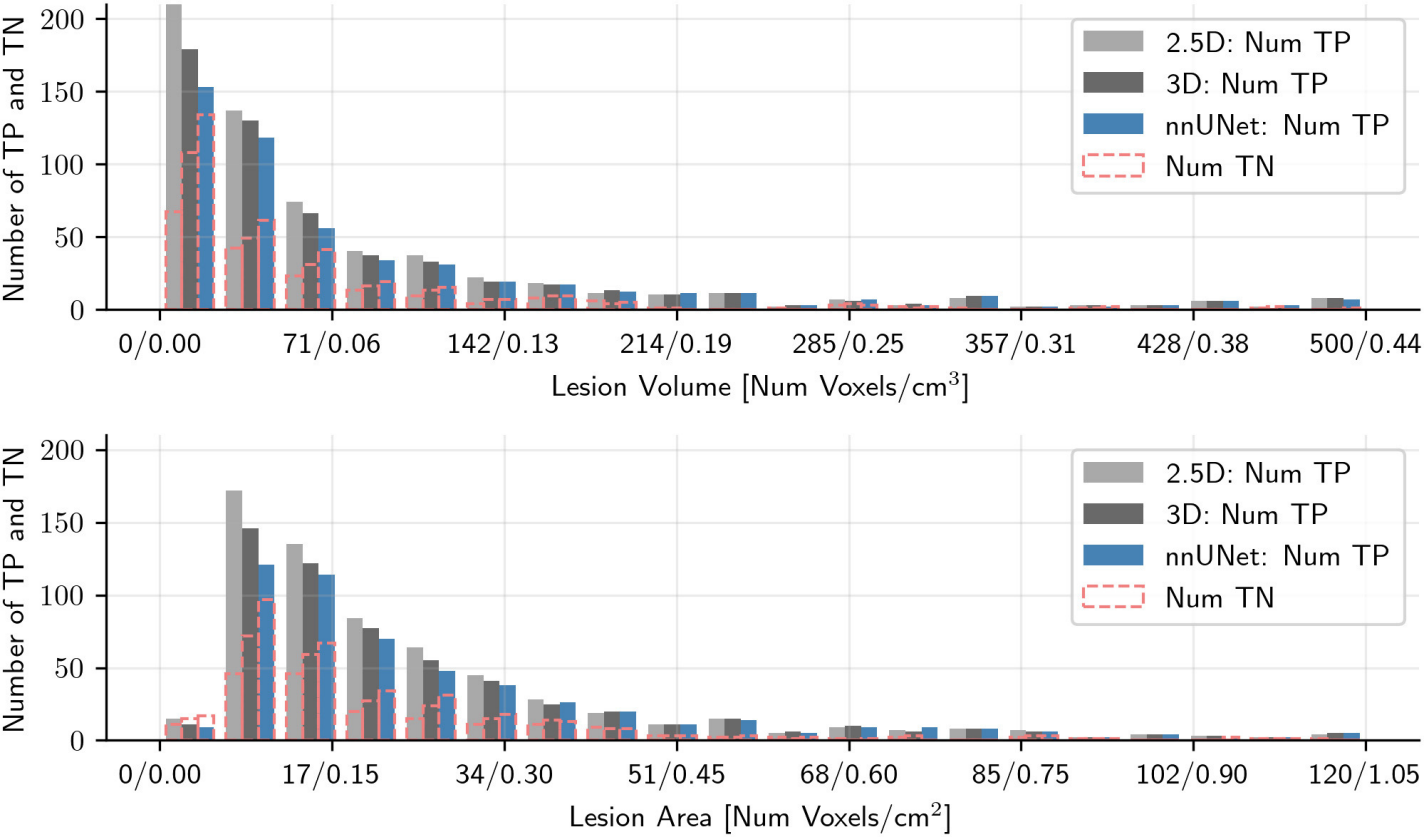
Histogram plots of the number of correctly predicted metastases and the number of missed metastases as a function of the maximum metastatic area in the axial plane and metastatic volume for the Stanford patient test cohort for all three models tested. Metastases larger than 120 (area) or 500 (volume) voxels were excluded to improve readability. TP denote true positive metastases, i.e., correctly predicted metastases, and TN denotes true negative metastases, i.e., not predicted metastases.

**Figure 4:**
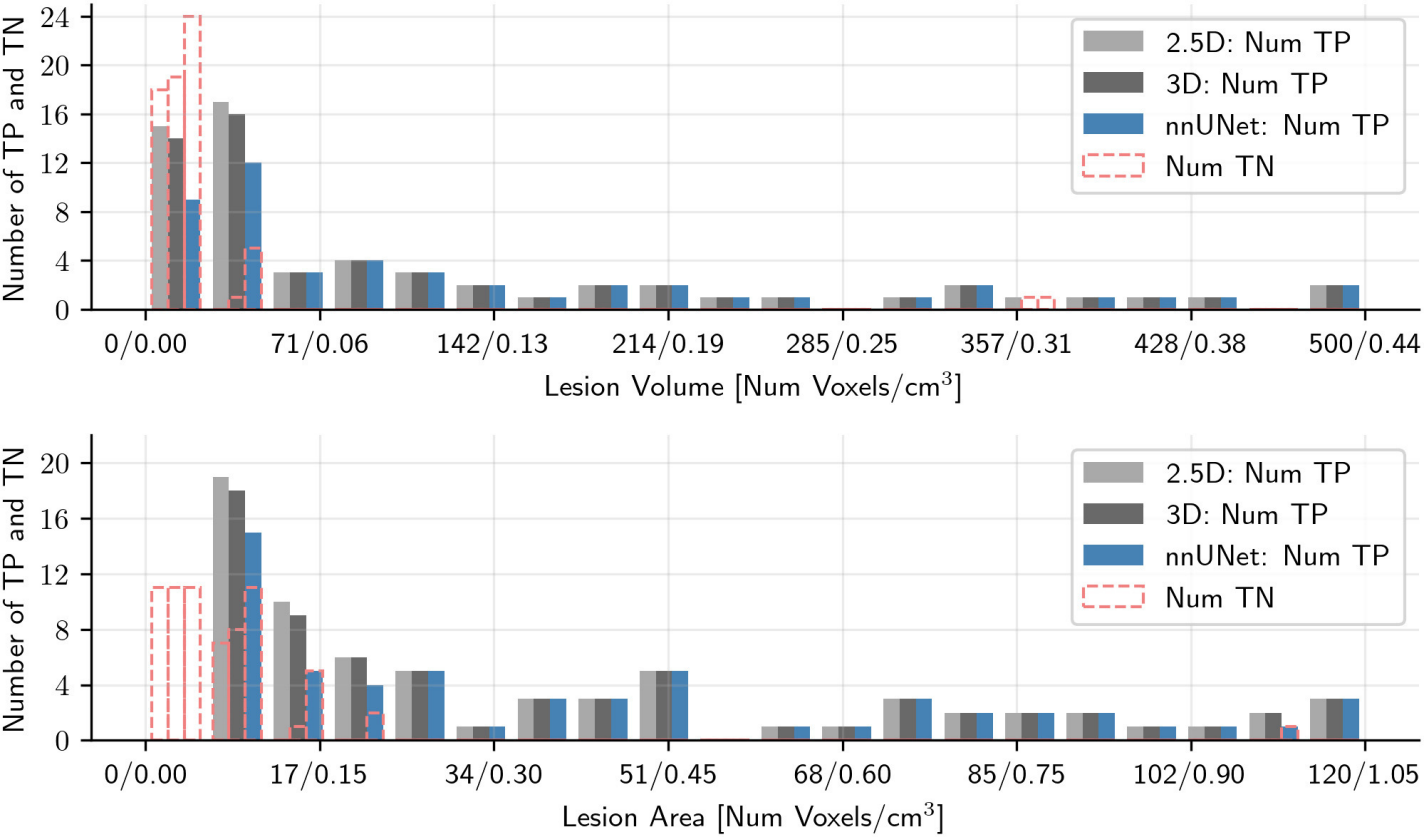
Histogram plots of the number of correctly predicted metastases and the number of missed metastases as a function of the maximum metastatic area in the axial plane and metastatic volume for the OUH cohort for all three models tested. Metastases larger than 120 (area) or 500 (volume) voxels were excluded to improve readability. TP denote true positive metastases, i.e., correctly predicted metastases, and TN denotes true negative metastases, i.e., not predicted metastases.

Figures 3, 4 show the number of correctly predicted and non-predicted metastases as a function of the metastatic volume and largest metastatic area in the Stanford and OUH cohorts, respectively. The largest non-detected metastatic volume was 0.53, 1.83, and 1.13 cm^3^; the largest non-detected axial area was 1.0, 2.0, and 1.4 cm^2^ on the Stanford cohort for the 2.5D, 3D, and nnU-Net, respectively. With respect to the OUH cohort, the largest non-detected metastatic volume was 0.018, 0.31, and 4.4 cm^3^; the largest non-detected axial area was 0.09, 1.1, and 3.7 cm^2^ for the 2.5D, 3D, and nn-UNet, respectively.

**Figure 5:**
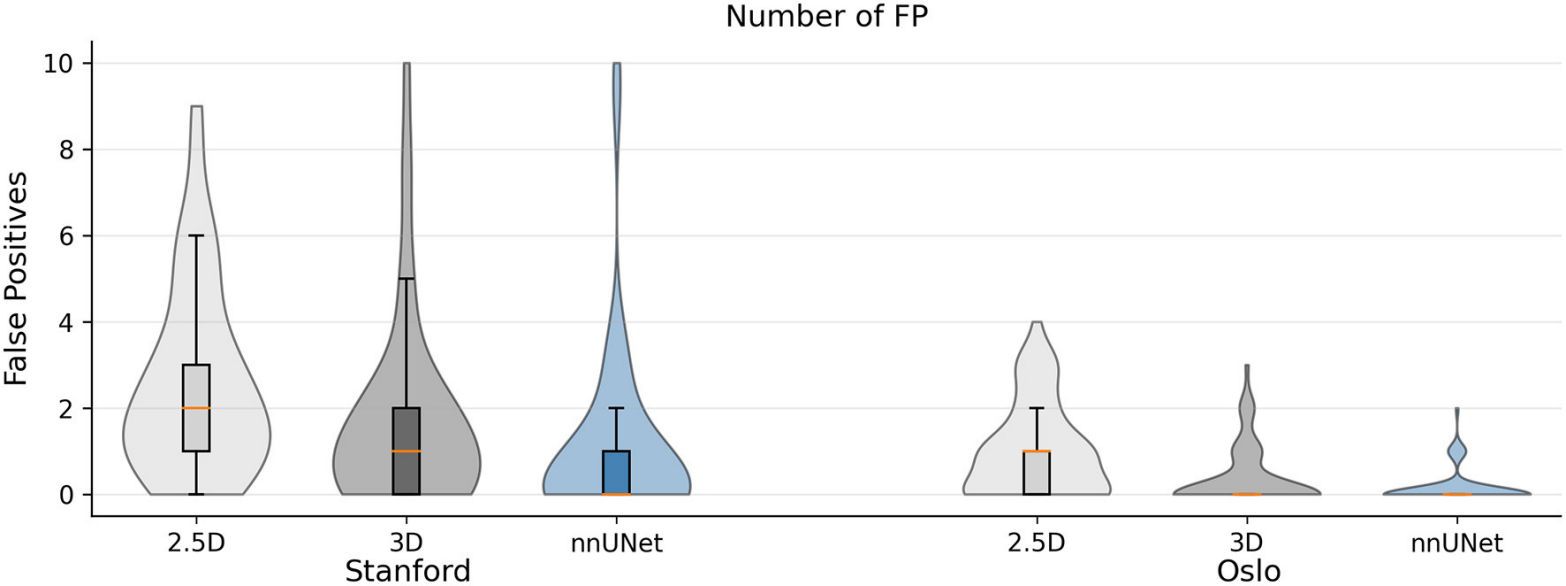
Violin plot of the number of false positives per patient for the Stanford and OUH cohorts for the 2.5D, 3D, and nnU-Net. Patients with more than 10 false positives were excluded to improve readability. This accounts to five patients in the Stanford cohort and zero patients in the OUH cohort. The median number of false positives were either 2, 1, or 0 in the Stanford cohort and 1 or 0 in the OUH cohort.

Violin plots of the false positive distribution for both cohorts are given in Figure 5. The number of false positives varied greatly between patients, and the maximum number of false positives for a single patient on the Stanford cohort was 62, 40, and 15; the maximum number of false positives for a single patient on the OUH cohort was 4, 3, and 2. The median number of false positives on the Stanford cohort was 2, 1, and 0 for the 2.5D, 3D, and nnU-Net. In the OUH cohort, both 3D models had a median number of false positives of 0, and the 2.5D network had a median number of 1. We note that the outlier patients greatly skew the false positive average value, and if the three patients with largest number of false positives were excluded, the average number of false positives per patient would be 3.6, 1.9, and 1.0 for the 2.5D, 3D, and nnU-Net, respectively.

**Figure 6:**
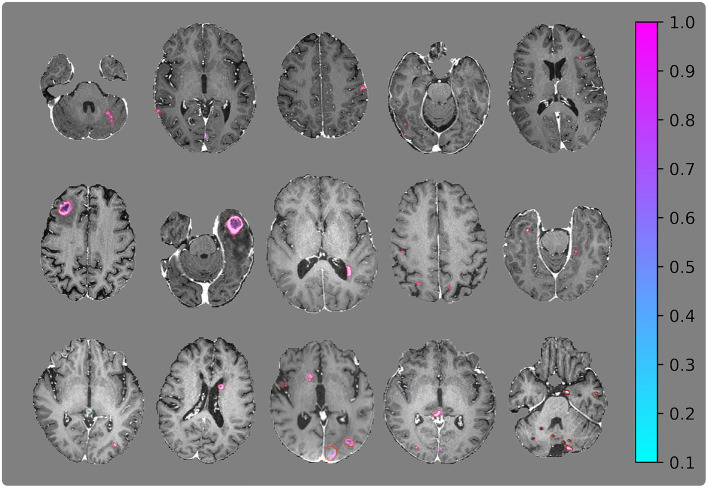
Visualization of segmentation examples from the Stanford cohort with the ground truth annotation (red regions of interests) and the segmentation probability map from the 2.5D network. The **(bottom right)** image slice belongs to the patient with 153 metastases and is one of the worst cases from the test set.

**Figure 7:**
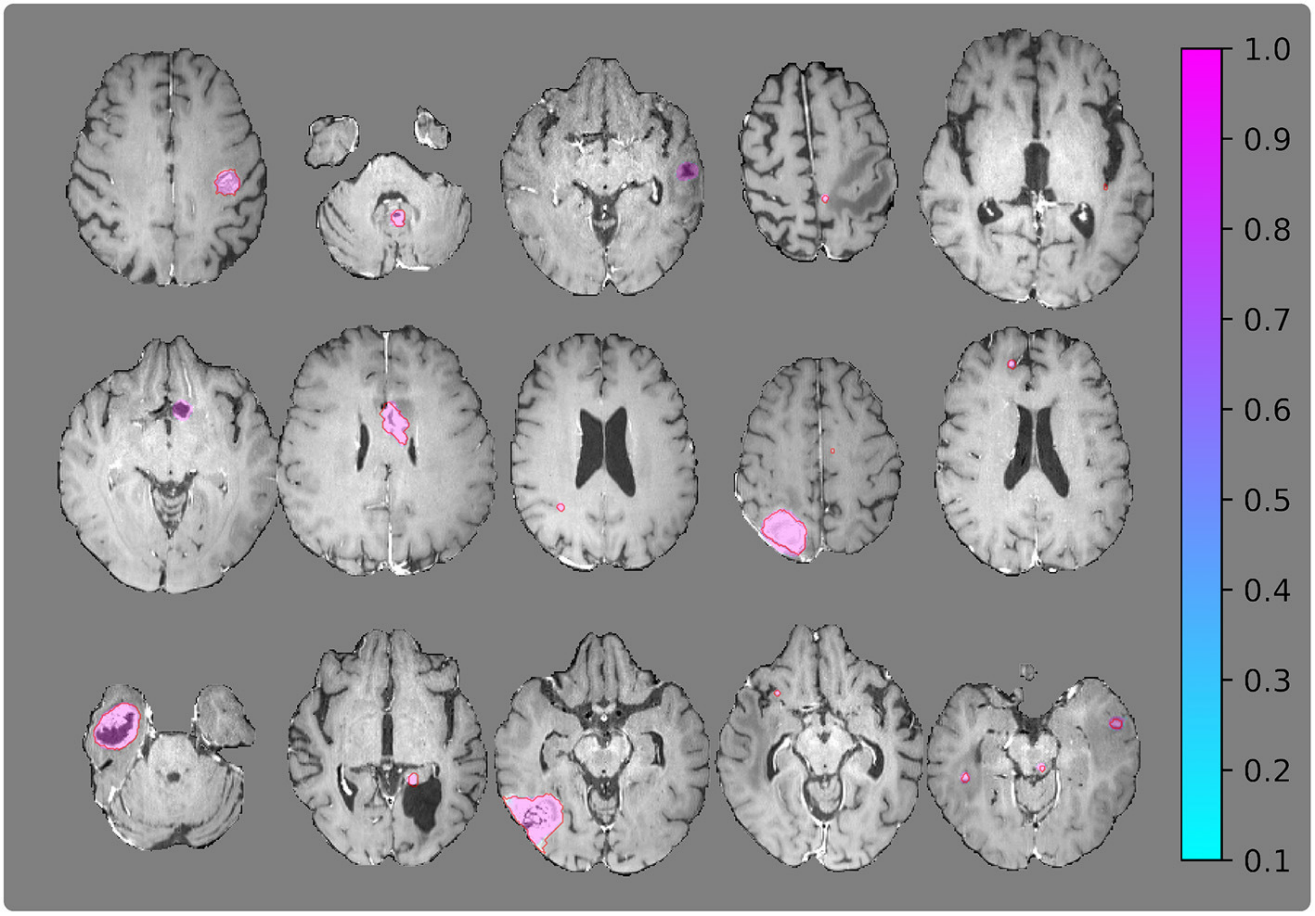
Visualization of segmentation examples from the OUH cohort with the ground truth annotation (red regions of interests) and the segmentation probability map from the 2.5D network.

Figures 6, 7 show the resulting probability maps for representative slices from the Stanford and OUH cohorts, respectively.

## 4. Discussion

In this work, we have tested and evaluated 2.5D, 3D, and nnU-Net for brain metastases segmentation. Our results suggest that all methods can successfully segment and detect brain metastases with few false positives on multinational data. To that end, we have developed robust deep learning segmentation models that can accurately segment brain metastases for a varying number of available MRI image-series: BRAVO, T1 pre/post contrast and FLAIR. Model evaluation was performed on multinational data from two large university hospitals, from which one cohort was not used during model training.

The results from Table 4 shows that nnU-Net predicts fewer false positives and have a slightly higher dice similarity score than the proposed 2.5D and 3D networks, but with a reduced overall sensitivity and a per patient sensitivity. This follows the design philosophy where true positives were deemed more important false positives resulting from the use of focal Tversky loss and upweighting of metastases slices. Nonetheless, nnU-Net provide accurate segmentation of brain metastases with few false positives.

The proposed 2.5D and 3D models showed robust segmentation performance and both models achieved a dice similarity coefficient of 0.84 and 0.93 for the Stanford and OUH cohorts, respectively. Moreover, the proposed models showed high accuracy: on the Stanford cohort the models detected 79% and 71% for the 2.5D and 3D model, respectively; on the OUH cohort the models detected 88% and 86% of all metastases for the 2.5D and 3D model, respectively. We note that the 2.5D network detected more metastases than its 3D counterpart on both cohorts, but with an increased false positive rate. This implies that a 2.5D network exhibits increased sensitivity, but with an increased false positive rate.

The models tested in this work performed considerably better on the OUH cohort compared to the Stanford cohort. This shows how different cohorts can affect model segmentation performance. It is reasonable to assume that this variation is caused by differences in MRI acquisitions, patient demographic and cohort-specific variations in the characteristics of the metastases. In general, the OUH cohort contained higher quality and more homogeneous T1-post contrast image-series. Moreover, since all patients in the OUH cohort were eligible for stereotactic radiotherapy, they had fewer and larger metastases, recently shown to provide better segmentation and prediction results (Grøvik et al., 2021). Like previous works for brain metastases segmentation (Charron et al., 2018; Bousabarah et al., 2020; Dikici et al., 2020; Grøvik et al., 2020, 2021; Zhang et al., 2020; Zhou et al., 2020a), we also noted a reduced segmentation performance for smaller metastases, with this trend being more pronounced for the OUH cohort. This can be seen from Figures 3, 4 where the ratio of missed/detected metastases was increased with decreasing lesion area. An interesting note is that this sentiment does seem to hold true for the metastases volume to the same degree. In general, we noted that the Stanford cohort contained more “challenging” cases and were less homogeneous when compared to OUH cohort. This difference in homogeneity is due to all OUH patients were to receive stereotactic radiotherapy, which was not case for the Stanford cohort.

Compared to previous works on the same Stanford cohort (Grøvik et al., 2020) and OUH cohort (Grøvik et al., 2021; Yi et al., 2021), our model archives a similar or improved per patient detection sensitivity with 0.88/0.83 compared to their previously published average sensitivity of 0.83, whilst reducing the total number of false positives from an average of 8.3 to 6.2/3.2 for the 2.5D and 3D networks, respectively. A similar, but more pronounced effect can be seen for the OUH cohort where the number of false positives was reduced from an average of 12.3 (Grøvik et al., 2021) to 1.0 or 0.4 per patient for the 2.5D and 3D networks, respectively. From this, it can be concluded that the model proposed in this study produces less false positives when compared to previous work, whilst achieving similar or improved sensitivity.

A direct inter-study comparison of segmentation performance in recent studies is questionable due to data variations, as is evident from this study. Still, the model achieves performance comparable to recent studies, with the relative low sensitivity on the Stanford cohort being a likely result from the large number of small metastases and the inhomogeneous nature of the patient cohort due to not having any inclusion criteria. Nonetheless, the number of false positives reported for the OUH cohort is among the lowest reported in literature at the time of writing (Zhou et al., 2020b; Hsu et al., 2021; Jünger et al., 2021; Pennig et al., 2021; Rudie et al., 2021). Still, we note the relative low sensitivity for the Stanford cohort in contrast to other work that have reported higher sensitivity (Xue et al., 2020; Cho et al., 2021). Further improvements would require additional training data to combat model overfitting during training.

Although this study has shown that the proposed method can generalize to multinational data, additional independent data from other sites would be necessary before endorsing clinical use and it would further strengthen this works claims that the model can generalize across multiple institutions. We recommend the 3D variant due to the drastic reduction in false positives compared to the 2.5D variant while maintaining a good sensitivity.

## 5. Conclusion

This study presents multiple models that can detect and segment brain metastases on multinational MRI data with high accuracy and a reduced number of false positive predictions compared to previous studies. Still, robust segmentation of very small metastases remains a challenge.

## Data availability statement

Publicly available datasets were analyzed in this study. The Stanford cohort is available here: https://aimi.stanford.edu/brainmetshare. The OUH dataset will be made publicly available upon completion of the clinical study. In the interim, the data is available from the corresponding author upon reasonable request.

## Ethics statement

The studies involving human participants were reviewed and approved by Regional Medical Ethics Committee for Oslo University Hospital and the Institutional Review Board at Stanford University. The patients/participants from OUH provided their written informed consent to participate in this study, and Stanford Review Board waived the requirement for informed consent for the Stanford cohort.

## Author contributions

JO, EG, DY, KE, DR, and GZ contributed to the design and implementation of the research. MI, ET, AL, CS, KJ, ÅH, KE, and GZ made substantial contribution to the acquisition of data. JO, EG, and DY organized and preprocessed the data. JO designed the computational setup, integrated the deep neural network for training, testing and analysis, and wrote the manuscript while AB revised it critically for important intellectual content. All authors contributed to the article and approved the submitted version.

## Footnotes

^1^ https://github.com/JonOttesen/Met-Seg
